# The association between low glucose-6-phosphate dehydrogenase activity level and hepatitis B virus infection among pre-pregnant reproductive-age Chinese females

**DOI:** 10.1038/s41598-019-40354-7

**Published:** 2019-03-07

**Authors:** Jun Zhao, Xu Zhang, Ting Guan, Qiaoyun Dai, Wenshan He, Hongguang Zhang, Yuanyuan Wang, Bei Wang, Zuoqi Peng, Xuhuai Hu, Daxun Qi, Xueying Yang, Yue Zhang, Xu Ma

**Affiliations:** 1 National Research Institute for Family Planning, Beijing, China; 2 National Human Genetic Resources Center, Beijing, China; 3 Department of medical record management, The Affiliated YanAn Hospital of Kunming Medical University, Yunnan, China; 4 Shenzhen Health Development Research Center, Guangdong, China; 5 School of Public Health, Southeast University, Jiangsu, China; 6 Graduate School of Peking Union Medical College, Beijing, China

## Abstract

The relationship between females with low glucose-6-phosphate dehydrogenase activity level (LG6PD) and HBV infection is unclear. We conducted a cross sectional study of 124 406 reproductive-age Chinese females who participated in the National Free Pre-conception Check-up Projects to investigate the risk of HBV infection among females with LG6PD and its effect on liver enzyme. Based on HBV serological test results, the participants were divided into the susceptible, immunized, and HBV infected groups. The multivariable-adjusted odds ratios (ORs) for HBV infection in LG6PD participants were 1.71 (95% confidence interval (CI): 1.45–2.01) and 1.41 (95% CI: 1.23–1.62), respectively with the susceptible and immunized participants as references, compared to those without LG6PD. Participants with HBV infection only and combined with HBV infection and LG6PD had 184% and 249% significantly higher risks of elevated alanine transaminase (ALT) (susceptible participants as reference). If the immunized participants were used as reference, significant higher odds of elevated ALT occurred (3.48 (95% CI: 3.18–3.80), 4.28 (95% CI: 2.92–6.28)). Thus, reproductive-age females with LG6PD had a higher prevalence of HBV infection, and LG6PD might exacerbate ALT elevation in HBV infected females. Our findings underscore the need to explore collaborative management approaches for these two diseases among reproductive-age females for maternal and child health.

## Introduction

Low glucose-6-phosphate dehydrogenase activity level (LG6PD) is one of typical features of G6PD deficiency, which is an X-linked incomplete dominant inheritance of red blood cell enzyme disease, as well as one of the most common causes of hemolytic anemia^1,2^. Reports indicate that more than 400 million people are affected by G6PD deficiency worldwide^1^. According to the population monitoring results of 2016 annual Chinese maternal and child health monitoring report, G6PD deficiency ranked second highest among all birth defects^3^. Most provinces in China had included the G6PD enzyme activity test program in neonatal screening. Previous studies showed that LG6PD was associated with reduced nicotinamide adenine dinucleotide phosphate (NADPH), which is an important antioxidant in humans^4^. Low levels of NADPH bring about oxidative stress disorder which could result in dysfunctional granulocytes^5^ and thus enhance the susceptibility to viral infections^2,6,7^.

Hepatitis B virus (HBV) infection and its related complications are a primary public health threat worldwide^8,9^. An estimated 2 billion people have been infected with HBV globally, and more than 257 million are chronic carriers of the virus^10,11^. China has a huge population base (1374 million in 2015)^12^ and a continuing burden of HBV infection (with about 90 million hepatitis B surface antigen (HBsAg) positive (HBsAg(+)) people)^13^, which is known as the primary driver of the global prevalence of HBV infection^9,13^.

A previous population study suggested that individuals with G6PD deficiency were twice as likely to acquire HBV infection as those without G6PD deficiency^14^. However, Chen *et al*.^7^ suggested that G6PD might be an endogenous promoting factor for HBV replication, which participates in and influences the replication process through interferon stimulated gene pathways. Considering the mother-to-child transmission is one of the main transmission routes of HBV, and the liver burden of HBV infected females could be aggravated during their pregnancy period, furthermore, the risk of spontaneous abortion, preterm labor and other adverse pregnancy outcomes might also increase^15–17^. Therefore, it is necessary to study the role of LG6PD in HBV infection and its effect on liver enzyme among pre-pregnant reproductive-age females to improve the health of both mothers and children.

Shenzhen is a city located in the southern coast of Guangdong province, China, where the prevalence of G6PD deficiency (1.14~3.68%^18,19^) and HBV infection (7.1% HBsAg(+)^20^) are both high in female population. Therefore, we carried out a cross sectional study to investigate the relationship between LG6PD and HBV infection in Shenzhen among reproductive-age females who participated in the National Free Pre-conception Check-up Projects (NFPCP), which is a national health service supported by the Chinese government^21^.

The primary objective of this study was to test the hypothesis that reproductive-age females with LG6PD have a higher prevalence of HBV infection. The secondary objective was to explore the effect influence of LG6PD on liver enzyme among HBV infected females.

## Methods

### Participants and study design

The NFPCP is a nationwide, population-based cross-sectional study project aimed at providing free pre-pregnancy medical examinations for reproductive-age couples who have made their conception plan in 6 months^22^. The overall goal of the project is to reduce the incidence of adverse pregnancy outcomes throughout the country. Trained local health professionals use a questionnaire-based survey to complete a standardized family health file for each participating couple, and also complete medical examinations. Completed files are then converted into electronic records and transferred into the NFPCP medical service information system for storage. This information system was developed by the National Research Institute for Family Planning, and was built with logic checks and instrument interfaces to avoid human errors. Prior to the submission, the data would be rechecked thoroughly.

All medical institutions involved in the NFPCP in Shenzhen have been included in the G6PD enzyme activity screening program since 2013. In order to ensure reliability of the serology examinations, activities in all sites are implemented with standard operating procedures and subject to the annual national external quality assessment. The detailed study design and organization of this project were described elsewhere^23^.

**Figure 1:**
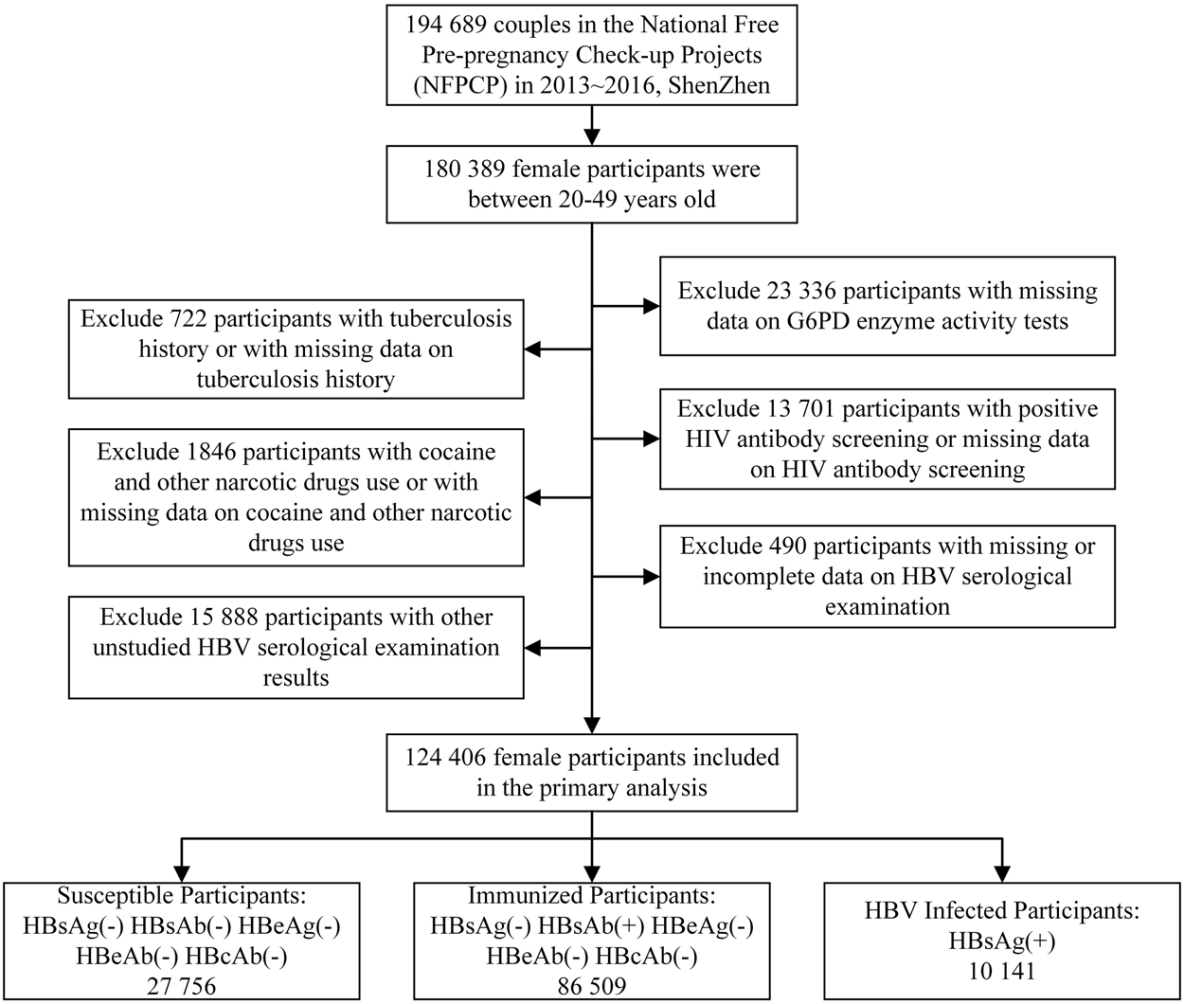
Flowchart of Study Population.

This study was an original research based on the NFPCP and the data of 22 sites used were obtained from the NFPCP system. From January 1, 2013 to December 31, 2016, comprising 180 389 eligible females aged 20–49 participated in the NFPCP in Shenzhen, China. Participants with missing data on G6PD enzyme activity, HBV serological examination, tuberculosis history, human immunodeficiency virus (HIV) antibody screening and use of cocaine and other narcotic drugs, as well as those with tuberculosis history or positive HIV antibody screening or cocaine and other narcotic drugs use were excluded. Finally, after excluding other HBV serological test results not intended for this study, data on 124 406 participants were included in the analysis, and they were divided into three categories including the susceptible, immunized, and HBV infected participants, according to the HBV serological test results. Susceptible participants were defined as being HBsAg negative (HBsAg(−)) and hepatitis B surface antibody negative (HBsAb(−)) and hepatitis B e-antigen negative (HBeAg(−)) and hepatitis B e-antibody negative (HBeAb(−)) and hepatitis B core-antibody negative (HBcAb(−)). Immunized participants were defined as being HBsAg(−) and HBsAb positive (HBsAb(+)) and HBeAg(−) and HBeAb(−) and HBcAb(−). HBV infected participants were defined as being HBsAg(+). The susceptible and immunized participants were selected as control groups separately while the HBV infected participants were used as the case group (detailed information of the study population was shown in Fig. 1**)**.

### Data collection

The standardized questionnaires were completed through face-to-face interviews while medical examinations were conducted by trained health professionals. Demographic characteristics and lifestyle information were collected by questionnaires from the husband and the wife, separately. The variables included were date of birth, nationality, rural/urban inhabitant, migrant population based on the address of household registration, educational level, alcohol consumption, smoke exposure (active or passive smoking), and self-reported history of hepatitis B vaccination. Body weight and height were measured with light indoor clothes without shoes and other accessories based on a standardized protocol. G6PD enzyme activity of erythrocytes, serologic HBV markers, and alanine transaminase (ALT) levels were tested using blood samples collected after at least 8 hours fasting, using ethylenediamine tetra-acetic acid and heparin lithium-anticoagulant vacuum tubes. The samples were stored at 4 °C in the freezers and were analyzed within 24 hours. G6PD enzyme activity was tested by a standardized quantitative spectrophotometric analysis. Serologic HBV markers including HBsAg, HBsAb, HBeAg, HBeAb, and HBcAb were tested by enzyme-linked immune-sorbent assay.

This study was approved by the Institutional Research Review Board at the National Health Commission and was conducted in accordance with the Declaration of Helsinki. All participants signed written informed consent prior to their participation.

### Definition of variables

Variables in this study included demographic characteristics, lifestyle information and serological test results. Age was split into three groups (20–29, 30–39, and 40–49 years old), while nationality was categorized as ‘Han’ and ‘others’. Educational level was categorized into two groups (bachelor degree or above, high school or below). The migrant population was defined as non-permanent population of Guangdong province. Body mass index (BMI) was calculated using weight and height based on the following formula: BMI = weight/height^2^ (kg/m^2^), and then was categorized into four groups as follows: underweight (<18.5 kg/m^2^), normal weight (18.5–23.9 kg/m^2^), overweight (24.0–27.9 kg/m^2^), and obesity (≥28.0 kg/m^2^). LG6PD was defined as G6PD enzyme activity below the lower limit of medical reference range based on the quantitative test results (Appendix 1). In this study, husbands with HBsAg(+) were defined as husband’s HBV infection.

### Statistical analysis

The variables included in this study on the characteristics of the participants in the three groups were presented as count (percentages). *χ*^2^ tests were used for categorical variables among the case group and two control groups. We used logistic regression to estimate the odds ratios (ORs) and their corresponding 95% confidence intervals (CIs) for the association between LG6PD and HBV infection by comparing the HBV infected participants with the susceptible and immunized participants, respectively. In addition to the wife’s own variables, literature has been reported that spouses of HBsAg(+) patients have a higher risk of HBV infection than spouses of HBsAg(−) patients, and there is a higher risk of HBV transmission between spouses^24^, so the multivariable-adjusted logistic regression was employed to adjust for wife’s demographic characteristics, lifestyle information, BMI, as well as husband’s HBV infection.

Since HBeAg can to some extent indicate the level of HBV infectiousness^23^, we further divided HBV infected participants into two groups (HBsAg(+) HBeAg(−) and HBsAg(+) HBeAg(+)) and compared them with the susceptible and immunized participants separately using multivariable logistic regression, to further identify whether reproductive-age females with LG6PD have a higher prevalence of HBV infection.

To further explore the effect of LG6PD on liver enzyme of participants with HBV infection, we examined ALT elevation as the outcome variable. Participants were divided into four groups according to LG6PD and HBV infection statuses, respectively among the HBV infected and susceptible participants, as well as among the HBV infected and immunized participants. These four groups were: 1) participants with neither HBV infection nor LG6PD (HBsAg(−) LG6PD(−)), 2) participants without HBV infection but with LG6PD (HBsAg(−) LG6PD(+)), 3) participants with HBV infection but without LG6PD (HBsAg(+) LG6PD(−)), and 4) participants with HBV infection combined with LG6PD (HBsAg(+) LG6PD(+)). Participants with HBsAg(−) LG6PD(−) were selected (among the four groups) as the reference group for comparison. The ORs and 95% CIs were determined to assess the impact of LG6PD on ALT elevation in HBV infected participants. Additionally, we compared the risk of different levels of ALT elevation (slight (ALT ≥ 45 U/L) and moderate (ALT ≥ 60 U/L)) in these four groups.

Subgroup analyses were conducted according to demographic characteristics, lifestyle information, BMI and husband’s HBV infection. Statistical analyses were conducted using Microsoft R Open, version 3.3.3 (Microsoft, Inc, Redmond, WA, USA).

## Results

### Characteristics of the study population according to HBV infection status

By December 31, 2016, 124 406 eligible females aged 20–49 participated in the NFPCP in Shenzhen, China. The prevalence of LG6PD among the participants was 2.12%. The characteristics of the study population were presented according to HBV infection status **(**Table 1**)**. Compared with the susceptible participants, HBV infected participants were more likely to be of Han nationality, urban inhabitants, with higher educational level, lower BMIs, and have more smoke exposure, while compared with the immunized participants, they were more likely to be rural inhabitants, with lower educational level, greater BMIs, and have less smoke exposure. Furthermore, compared with the susceptible or immunized groups, HBV infected participants were more likely to be older, resident population, no alcohol consumption, less HBV vaccination injection and with more husband’s HBV infection. In particular, the prevalence of LG6PD was significantly higher in the HBV infected group (Table 1).

**Table 1:** Characteristics of study population according to HBV infection status.

Variables	HBV Infected Participants, *N*(%)	Susceptible Participants		Immunized Participants	
		*N*(%)	*P*	*N*(%)	*P*
Total	10 141 (100.00)	27 756 (100.00)	…	86 509 (100.00)	…
Age, y			<0.0001		<0.0001
20–29	6 161 (60.75)	19 184 (69.12)		56 021 (64.76)	
30–39	3 672 (36.21)	8 002 (28.83)		28 607 (33.07)	
40–49	308 (3.04)	570 (2.05)		1 881 (2.17)	
Nationality			<0.0001		0.1396
Han	9 830 (97.19)	26 436 (95.56)		83 656 (96.92)	
Others	284 (2.81)	1 229 (4.44)		2 659 (3.08)	
Rural/urban inhabitant			<0.0001		<0.0001
Rural	6 039 (59.55)	19 063 (68.68)		44 699 (51.67)	
Urban	4 102 (40.45)	8 693 (31.32)		41 810 (48.33)	
Migrant population			<0.0001		<0.05
Yes	3 339 (32.93)	14 277 (51.44)		29 390 (33.97)	
No	6 802 (67.07)	13 479 (48.56)		57 119 (66.03)	
Educational level			<0.0001		<0.0001
Bachelor degree or above	6 074 (60.45)	13 886 (50.50)		59 746 (69.56)	
High school or below	3 974 (39.55)	13 611 (49.50)		26 144 (30.44)	
Body mass index, kg/m^2^			<0.0001		<0.01
Underweight (<18.5)	2 098 (21.10)	5 362 (19.69)		18 006 (21.19)	
Normal weight (18.5−)	6 723 (67.62)	18 233 (66.96)		57 900 (68.13)	
Overweight (24.0−)	913 (9.18)	2 943 (10.81)		7 672 (9.03)	
Obesity (≥28.0)	209 (2.10)	691 (2.54)		1 401 (1.65)	
Alcohol consumption			<0.0001		<0.0001
Yes	1 654 (16.35)	5 603 (20.25)		18 610 (21.58)	
No	8 462 (83.65)	22 068 (79.75)		67 624 (78.42)	
Smoke exposure			<0.001		<0.001
Yes	3 580 (35.30)	9 223 (33.23)		32 072 (37.07)	
No	6 561 (64.70)	18 530 (66.77)		54 434 (62.93)	
Self-reported history of hepatitis B vaccination			<0.0001		<0.0001
Yes	2 665 (26.43)	10 804 (39.10)		51 040 (59.21)	
No	7 418 (73.57)	16 830 (60.90)		35 157 (40.79)	
Husband’s HBV infection			<0.0001		<0.0001
Yes	1 399 (16.14)	1 771 (7.86)		8 875 (12.02)	
No	7 270 (83.86)	20 750 (92.14)		64 973 (87.98)	
Low G6PD activity level		<0.0001			<0.0001
Yes	328 (3.23)	494 (1.78)		1 728 (2.00)	
No	9 813 (96.77)	27 262 (98.22)		84 781 (98.00)	

N, number; HBV, hepatitis B virus; G6PD, glucose-6-phosphate dehydrogenase.

### Association between low G6PD activity level and HBV infection

Among the participants, prevalence of LG6PD were 3.23%, 1.78%, 2.00% in the HBV infected, susceptible control, and immunized control groups, respectively. The ORs of LG6PD with HBV infection were presented in Table 2. In the HBsAg(+) and susceptible control groups, participants with LG6PD had a 71% higher risk of HBV infection (multivariable-adjusted OR: 1.71, 95% CI: 1.45–2.01) than those without LG6PD. In the HBsAg(+) and immunized control groups, compared with participants without LG6PD, those with LG6PD had a 41% higher risk of HBV infection (multivariable-adjusted OR: 1.41, 95% CI: 1.23–1.62).

**Table 2:** Association between low G6PD activity level and HBV infection.

Low G6PD activity level	Susceptible participants vs HBV infected participants			Immunized participants vs HBV infected participants		
	Crude *OR* (95% *CI*)	Husband’s HBV infection Adjusted^†^ *OR* (95% *CI*)	Multivariable-Adjusted^‡^ *OR* (95% *CI*)	Crude *OR* (95% *CI*)	Husband’s HBV infection Adjusted^†^ *OR* (95% *CI*)	Multivariable-Adjusted^‡^ *OR* (95% *CI*)
No	Ref	Ref	Ref	Ref	Ref	Ref
Yes	1.84 (1.60–2.12)	1.81 (1.55–2.11)	1.71 (1.45–2.01)	1.64 (1.45–1.85)	1.60 (1.41–1.82)	1.41 (1.23–1.62)

OR, odds ratio; CI, confidence interval; HBV, hepatitis B virus; G6PD, glucose-6-phosphate dehydrogenase; Ref, reference.

^**†**^Husband’s HBV infection Adjusted OR was adjusted for husband’s HBV infection. ^‡^Multivariable-Adjusted OR was adjusted for age, nationality, rural/urban inhabitants, migrant population, educational level, BMI, alcohol consumption, smoke exposure, self-reported history of hepatitis B vaccination, husband’s HBV infection.

We further divided HBV infected participants into two groups and found that in the susceptible control group and participants with HBsAg(+) HBeAg(−), the crude OR, husband’s HBV infection adjusted OR and multivariable-adjusted OR for HBV infection in participants with LG6PD were 1.79 (95% CI: 1.53–2.09), 1.81 (95% CI: 1.52–2.14), and 1.75 (95% CI: 1.46–2.09), respectively compared to participants without LG6PD. In the susceptible control group and participants with HBsAg(+) HBeAg(+), the crude and adjusted ORs were 2.02 (95% CI: 1.60–2.54), 1.81 (95% CI: 1.40–2.35), and 1.58 (95% CI: 1.21–2.08), respectively. In the immunized control and HBV infected groups, multivariable-adjusted ORs for HBV infection in participants with LG6PD were 1.44 (95% CI: 1.23–1.67) and 1.32 (95% CI: 1.02–1.71), respectively compared to those without LG6PD (Table 3).

**Table 3:** Association between low G6PD activity level and HBV infection according to HBeAg status.

Low G6PD activity level	HBV infected participants and Susceptible participants			HBV infected participants and Immunized participants		
	Crude *OR* (95% *CI*)	Husband’s HBV infection Adjusted^†^ *OR* (95% *CI*)	Multivariable- Adjusted^‡^ *OR* (95% *CI*)	Crude *OR* (95% *CI*)	Husband’s HBV infection Adjusted^†^ *OR* (95% *CI*)	Multivariable- Adjusted^‡^ *OR* (95% *CI*)
	HBsAg (+) HBeAg(−) vs Susceptible Participants			HBsAg (+) HBeAg(−) vs Immunized participants		
No	Ref	Ref	Ref	Ref	Ref	Ref
Yes	1.79 (1.53–2.09)	1.81 (1.52–2.14)	1.75 (1.46–2.09)	1.59 (1.39–1.82)	1.60 (1.38–1.85)	1.44 (1.23–1.67)
	HBsAg (+) HBeAg(+) vs Susceptible Participants			HBsAg (+) HBeAg(+) vs Immunized participants		
No	Ref	Ref	Ref	Ref	Ref	Ref
Yes	2.02 (1.60–2.54)	1.81 (1.40–2.35)	1.58 (1.21–2.08)	1.79 (1.44–2.23)	1.60 (1.25–2.05)	1.32 (1.02–1.71)

OR, odds ratio; CI, confidence interval; HBV, hepatitis B virus; HBeAg, hepatitis B e-antigen; G6PD, Glucose-6-phosphate dehydrogenase; Ref, reference.

^†^Husband’s HBV infection Adjusted OR was adjusted for husband’s HBV infection. ^‡^Multivariable-Adjusted OR was adjusted for age, nationality, rural/urban inhabitants, migrant population, educational level, BMI, alcohol consumption, smoke exposure, self-reported history of hepatitis B vaccination, husband’s HBV infection.

### Odds ratios for elevated ALT according to low G6PD activity level and HBV infection statuses

Table 4 showed the ORs and their corresponding 95% CIs for elevated ALT according to LG6PD and HBV infection statuses. Among 37 883 pre-pregnant reproductive-age females in the HBV infected and susceptible control groups, 1 496 (3.95%) showed a slight ALT elevation (ALT ≥ 45 U/L). Compared with participants with HBsAg(−) LG6PD(−), participants with HBsAg(+) LG6PD(−) had 184% higher risk of elevated ALT while those with HBsAg(+)  LG6PD(+) had 249% higher risk of elevated ALT **(**Table 4**)**, and the trend was significant (*χ*_trend_² = 418.35, *df* = 1, p < 0.0001). Among 96 627 pre-pregnant reproductive-age females in the HBV infected and immunized control groups, 2 672 (2.77%) showed slight ALT elevation. Compared with the HBsAg(−) LG6PD(−) group, the ORs for slight ALT elevation were 1.26 (95% CI: 0.94–1.69), 3.48 (95% CI: 3.18–3.80) and 4.28 (95% CI: 2.92–6.28), respectively in the HBsAg(−) LG6PD(+), HBsAg(+) LG6PD(−), and HBsAg(+) LG6PD(+) groups. The results were consistent with the susceptible control group used as a reference, but with higher odds ratios. We further calculated the ORs for moderate ALT elevation (ALT ≥ 60 U/L) and the results were consistent in all statuses with more significant differences in statistics.

**Table 4:** Odds ratios for elevated ALT according to low G6PD activity level and HBV infection statuses.

	HBV infected participants and Susceptible participants			HBV infected participants and Immunized participants		
	normal ALT (*n*)	elevated ALT (*n*)	*OR* (95% *CI*)	normal ALT (*n*)	elevated ALT (*n*)	*OR* (95% *CI*)
ALT < 45 U/L vs ALT ≥ 45 U/L						
HBsAg(−) LG6PD(−)	26 512	737	Ref	82 881	1 878	Ref
HBsAg(−) LG6PD(+)	481	13	0.97 (0.56–1.69)	1 680	48	1.26 (0.94–1.69)
HBsAg(+) LG6PD(−)	9 095	717	2.84 (2.55–3.15)	9 095	717	3.48 (3.18–3.80)
HBsAg(+) LG6PD(+)	299	29	3.49 (2.37–5.14)	299	29	4.28 (2.92–6.28)
ALT < 45 U/L vs ALT ≥ 60 U/L						
HBsAg(−) LG6PD(−)	26 512	395	Ref	82 881	994	Ref
HBsAg(−) LG6PD(+)	481	8	1.12 (0.55–2.26)	1 680	26	1.29 (0.87–1.91)
HBsAg(+) LG6PD(−)	9 095	438	3.23 (2.82–3.71)	9 095	438	4.02 (3.58–4.50)
HBsAg(+) LG6PD(+)	299	20	4.49 (2.82–7.14)	299	20	5.58 (3.53–8.81)

N, number; OR, odds ratio; CI, confidence interval; HBV, hepatitis B virus; LG6PD, low Glucose-6-phosphate dehydrogenase activity level; Ref, reference.

### Subgroup analyses of the association between low G6PD activity level and HBV infection

**Figure 2:**
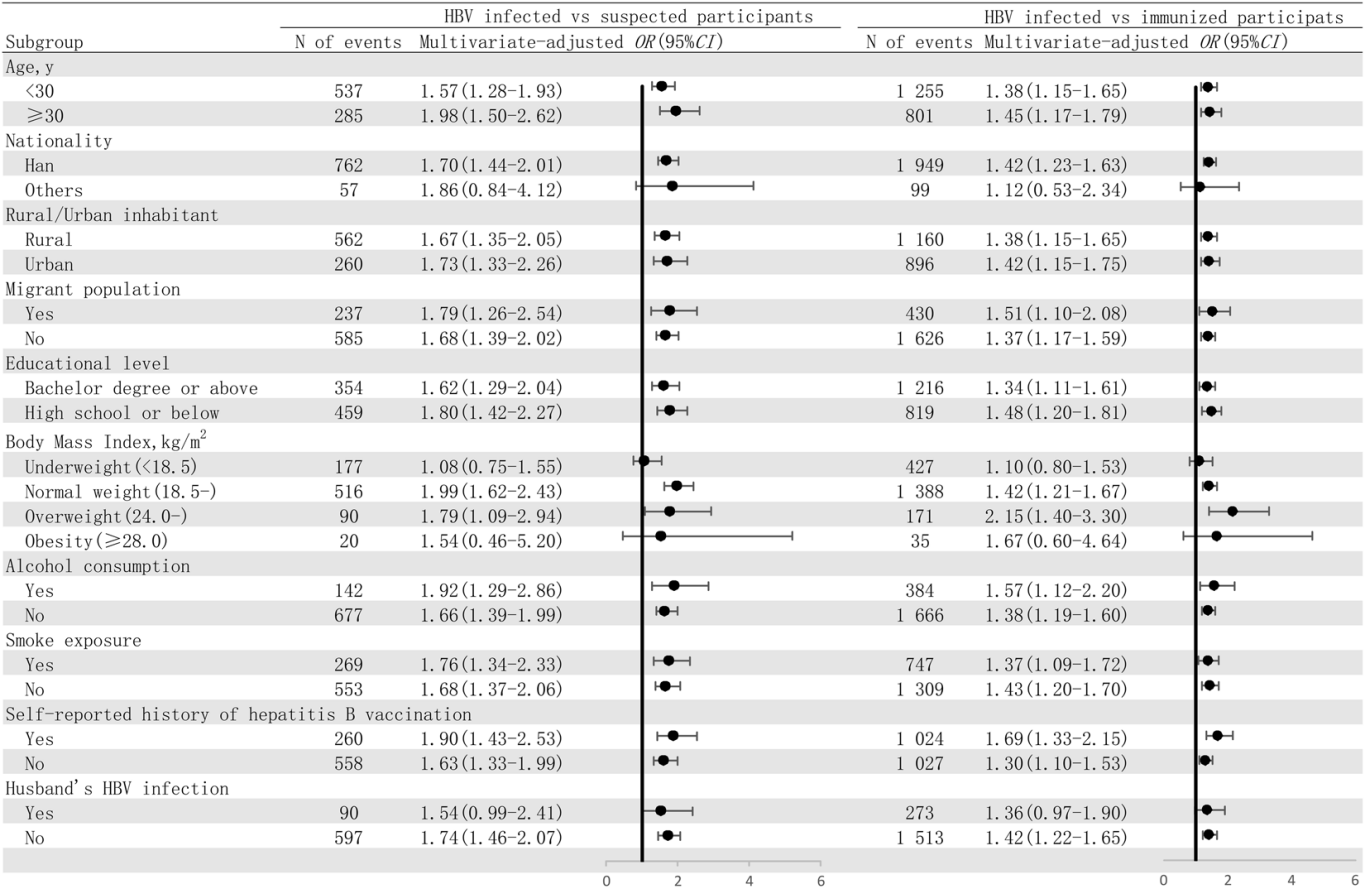
Subgroup analysis of the association between low G6PD activity level and HBV infection. N, number; OR, odds ratio; CI, confidence interval; HBV, hepatitis B virus; G6PD, Glucose-6-phosphate dehydrogenase. Note: In this figure N of events showed the count of LG6PD participants with HBV infection, and all of the subgroup analysis models were adjusted for age, nationality, rural/urban inhabitants, migrant population, higher educational level, BMI, alcohol consumption, smoke exposure, self-reported history of hepatitis B vaccination and husband’s HBV infection.

Results of subgroup analyses were shown in Fig. 2. We performed logistic regression analysis on age, nationality, urban/rural inhabitants, migrant population, educational level, BMI, alcohol consumption, smoke exposure, self-reported history of hepatitis B vaccination, and husband’s HBV infection separately. The results showed that multivariable-adjusted ORs in all subgroups were consistent whether we used the susceptible control group or immunized control group as reference.

## Discussion

G6PD deficiency is the most common metabolic disorder of red blood cells^25^, as well as the most widespread form of acute hemolytic anemia^26^, while HBV infection is a major mother-to-child transmission of disease with a heavy disease burden^8^. The WHO Working Group recommended that screening for G6PD deficiency in adults by quantitative estimation of the enzyme activity level in red cells, which was considered easy and desirable at the public health level^27^. In this large population-based study, we used this screening method to explore the relationship between LG6PD and HBV infection among pre-pregnant reproductive-age females. The results showed that females aged 20–49 with LG6PD had a higher risk of HBV infection and that LG6PD might exacerbate ALT elevation in HBV infected females.

Few studies have reported on the association between LG6PD (or G6PD deficiency) and HBV infection, but some findings were consistent with our results. A study from Ghana suggested that patients with viral hepatitis had twice the frequency of G6PD deficiency compared to the general population, and those with G6PD deficiency had a longer and more severe course of hepatitis^28^. Liu *et al*.^29^ found that the prevalence of LG6PD in patients with HBV infection in Guangzhou was 6.60% (20/303), while that of the control group was only 3.37% (20/593). Xiao *et al*.^14^ studied 10 626 objects on pre-marital physical examination, and found that those with G6PD deficiency were twice as likely to be infected with HBV as objects without G6PD deficiency, which demonstrated that G6PD deficiency could increase the risk of HBV infection. This study utilized data of a large population and found a significant association between LG6PD and HBV infection. It was interesting that the results on the susceptible control and HBV infected groups were similar to the results on the immunized control and HBV infected groups, but it was more evident when the susceptible control group was used as a reference.

Most researchers explained the potential mechanism by which LG6PD could result in the disorder of oxidative stress and hence, in the malfunctioning of granulocytes^30–33^. Some researchers^5,30^ suggested that LG6PD could reduce NADPH levels, thus decrease the production of nitric oxide, superoxide and hydrogen peroxide in leucocytes, and weaken the immune function thereof, thereby increase the susceptibility to pathogens. Wu *et al*.^6^ suggested that G6PD-knockdown cells could produce more reactive oxygen species, and high oxidative stress condition favors viral replication since it promotes viral gene expression. Other mechanism studies showed that LG6PD could affect the redox condition thereby affect the HSCARG-NF-κB (a newly identified nuclear factor κB) signaling axis, which could stimulate the expression of antiviral genes and induce susceptibility of the body cells to viruses^33^. Therefore, there are sufficient mechanisms reported in research findings to support the notion that LG6PD could enhance the susceptibility to virus infections.

In this study, after adjusting for covariate variables, the results showed that LG6PD could increase the risk of HBsAg(+) HBeAg(−) and HBsAg(+) HBeAg(+) regardless of whether the susceptible group or the immunized group was selected as reference, which indicated again that LG6PD might be a risk factor for HBV infection. However, multivariable-adjusted ORs for HBsAg(+) HBeAg(−) were greater than those of HBsAg(+) HBeAg(+), which was different from what we expected. Therefore, we further searched relevant literatures and found that there were indeed previous studies whose findings might be consistent with us. Chen *et al*.^7^ investigated the role of G6PD in HBV replication and the possible mechanism of action in liver tissues and cells, and found that G6PD might be an endogenous promoting factor for HBV replication by affecting the expression of genes related to the type I interferon pathway. Hu *et al*.^34^ put forward through research that G6PD suppression could lead to reduced concentrations of HBsAg and HBeAg in supernatant. Ding^35^ also reported that HBV infected cells were significantly lower at HBsAg, HBeAg and HBV DNA quantitative level after G6PD expression inhibited. However, there are few population studies on the association between LG6PD and HBV replication; thus, further studies are needed to investigate it.

We further calculated ORs for elevated ALT according to the four different statuses of LG6PD and HBV infection to explore the effect of LG6PD on liver enzyme of participants with HBV infection. It was found that LG6PD might exacerbate ALT elevation among HBV infected participants. Although no previous population studies have reported that LG6PD might exacerbate ALT elevation in people with HBV infection, some studies have suggested that LG6PD may influence the course and severity of hepatitis (other types of hepatitis other than hepatitis B). Through comparison of 18 cases of acute hepatitis A infection combined with G6PD deficiency and 18 cases of hepatitis A infection but with G6PD normal patients, Gotsman *et al*.^2^ suggested that G6PD deficiency might impede the repair of hepatocytes damaged by viral hepatitis as a result of increased oxidative damage to hepatocytes because of glutathione depletion, thus causing liver malfunction. Zheng *et al*.^36^ proposed that G6PD deficiency could aggravate hepatitis, and was prone to erythrocyte hemolysis which could result in liver damage. Another study^28^ suggested that prolonged jaundice from hepatitis could be attributed to G6PD deficiency. All of these studies might indirectly support the finding that LG6PD had effects on liver damage of patients with hepatitis.

We believe our research has important public health and clinical implications because G6PD deficiency and HBV infection and the related complications are primary public health threats worldwide^8,9^. Reproductive-age females with LG6PD combined with HBV infection are high risk population, especially during the pre-pregnancy and pregnancy periods. Therefore, effective interventions should be taken in this stage to ensure maternal and child health. Firstly, pregnancy could aggravate the liver burden in females with HBV infection and HBV infection could result in spontaneous abortion, preterm birth and other adverse outcomes^15–17^, while G6PD deficiency could result in spontaneous abortion, intrauterine fetal death, stillbirth, fetal abnormality and other adverse pregnancy outcomes^37,38^. Hence, liver enzyme monitoring, prenatal examination, and careful use of oxidative drugs (such as aspirin, phenacetin, and sulfadiazine) to avoid hemolytic anemia and increase liver cell damage^29^ should be strengthened for pregnant women who combined these two diseases. When these high-risk women are delivered, the newborns should also be administered with hepatitis B immunoglobulin early enough to effectively block mother-to-child transmission, and G6PD screening should also be performed simultaneously. Moreover, hepatitis B vaccination should be strengthened during childhood.

To our knowledge, our study was the first large-scale population study to explore the association between LG6PD and HBV infection in pre-pregnancy females in China, and the results in part provided some references of the association between these two diseases. However, some limitations should also be declared. First, due to the results of two screening methods of G6PD enzyme activity quantitative estimation (NADP + REDOX enzyme method and G6PD/6PGD ratio method) were similar^39^, we defined LG6PD as a categorical variable according to whether it was below the lower limit of medical reference range. Thus, we could not stratify G6PD activity levels into more specific groups for quantitative analysis. Second, instead of using HBV DNA to directly verify the association between LG6PD and HBV replication, we used HBeAg as an indicator to indirectly study the association between LG6PD and HBV infectiousness. Third, we only used once serological test result to define ALT elevation, other liver enzymes were not be measured in our study, and the data of whether the participants with HBV infection were receiving antiviral therapy was not collected completely. This might cause misclassification bias in the analysis of the effect of LG6PD on liver enzyme among HBV infected females. Lastly, the causal relationship between LG6PD and HBV infection could not be proved by a cross-sectional study. Prospective cohort studies and animal model studies should be conducted to further verify the association between the two diseases in future.

## Supplementary information

Table S1 The method and medical reference range for G6PD activity screening in all medical organizations included in this study.
