# Supplementary material for: The association between low glucose-6-phosphate dehydrogenase activity level and hepatitis B virus infection among pre-pregnant reproductive-age Chinese females
Source: Sci Rep. 2019 Mar 7;9:3865. doi: 10.1038/s41598-019-40354-7 (PMC6405931; doi:10.1038/s41598-019-40354-7)
Supplement: Supplementary file 1 — Table S1 The method and medical reference range for G6PD activity screening in all medical organizations included in this study. [file 41598_2019_40354_MOESM1_ESM.docx]

**The association between low glucose-6-phosphate dehydrogenase activity** **level** **and hepatitis B virus** **infection among** **pre-pregnant reproductive-age Chinese females**

Jun Zhao^1,2†^, Xu Zhang^3†^, Ting Guan^4^, Qiaoyun Dai^1,2^, Wenshan He^4^, Hongguang Zhang^1,2^, Yuanyuan Wang^1,2^, Bei Wang^5^, Zuoqi Peng^1,2^, Xuhuai Hu^4^ , Daxun Qi^1,2^, Xueying Yang^1,2^, Yue Zhang^1,6^, Xu Ma^1,2,6*^

^†^These authors contributed equally to this work.

**Affiliations:**

^1^ National Research Institute for Family Planning, Beijing, China;

^2^ National Human Genetic Resources Center, Beijing, China;

^3^ Department of medical record management, The Affiliated YanAn Hospital of Kunming Medical University, Yunnan, China;

^4^ Shenzhen Health Development Research Center, Guangdong, China;

^5^ School of Public Health, Southeast University, Jiangsu, China;

^6^ Graduate School of Peking Union Medical College, Beijing, China.

*Corresponding author: Xu Ma, MS, National Research Institute for Family Planning, National Human Genetic Resources Center, Graduate School of Peking Union Medical College, No. 12 Dahuisi Rd, Haidian District, Beijing 100081, China. Tel: +86 10 62179059. E-mails: genetic88@sina.com; [genetic88@126.com](mailto:genetic88@126.com).

## Appendix 1

**Table S1 The method and medical reference range for G6PD activity screening in all medical organizations included in this study.**

| **No.** | **Name of medical organizations** | **The screening method of G6PD activity** | **The medical reference range for G6PD activity screening** |
| --- | --- | --- | --- |
| 1 | Maternal and Child Care Service Center of Baoan District, Shenzhen, China | NADP+REDOX enzyme method | 1300-3600 U/L |
| 2 | Family Planning Service Center of Baoan District, Shenzhen, China | NADP+REDOX enzyme method | 1300-3600 U/L |
| 3 | Maternal and Child Care Service Center of Guangming New District, Shenzhen, China | NADP+REDOX enzyme method | 1300-3600 U/L |
| 4 | People′s Hospital of Guangming New District, Shenzhen, China | NADP+REDOX enzyme method | 1300-3600 U/L |
| 5 | Family Planning Service Center of Bantian Street, Shenzhen, China | NADP+REDOX enzyme method | 1300-3600 U/L |
| 6 | The Second People’s Hospital of Longgang District, Shenzhen, China | NADP+REDOX enzyme method | 1300-3600 U/L |
| 7 | The Third People’s Hospital of Longgang District, Shenzhen, China | NADP+REDOX enzyme method | 1300-3600 U/L |
| 8 | Central Hospital of Longhua New District, Shenzhen, China | NADP+REDOX enzyme method | 1300-3600 U/L |
| 9 | Maternal and Child Care Service Center of Futian District, Shenzhen, China | NADP+REDOX enzyme method | 1300-3600 U/L |
| 10 | Maternal and Child Care Service Center of Yantian District, Shenzhen, China | NADP+REDOX enzyme method | 1300-3600 U/L |
| 11 | People′s Hospital of Pingshan District, Shenzhen, China | NADP+REDOX enzyme method | 1300-3600 U/L |
| 12 | The Fourth People’s Hospital of Longgang District, Shenzhen, China | G6PD/6PGD ratio method | 0.70-1.90 |
| 13 | Family Planning Service Center of Longgang District, Shenzhen, China | G6PD/6PGD ratio method | 1.00-2.30 |
| 14 | Maternal and Child Care Service Center of Longhua New District, Shenzhen, China | G6PD/6PGD ratio method | 0.70-1.90 |
| 15 | People’s Hospital of Longhua New District, Shenzhen, China | G6PD/6PGD ratio method | 1.00-2.30 |
| 16 | People’s Hospital of Songgang Street, Shenzhen, China | G6PD/6PGD ratio method | 1.00-2.30 |
| 17 | Maternal and Child Care Service Center of Luohu District, Shenzhen, China | G6PD/6PGD ratio method | 1.00-2.30 |
| 18 | Maternal and Child Care Service Center of Nanshan District, Shenzhen, China | G6PD/6PGD ratio method | 1.00-2.30 |
| 19 | Family Planning Service Center of Luohu District, Shenzhen, China | G6PD/6PGD ratio method | 1.00-2.30 |
| 20 | Family Planning Service Center of Nanshan District, Shenzhen, China | G6PD/6PGD ratio method | 1.00-2.30 |
| 21 | Maternal and Child Care Service Center of Pingshan District, Shenzhen, China | G6PD/6PGD ratio method | 1.00-2.30 |
| 22 | Maternal and Child Care Service Center of Dapeng New District, Shenzhen, China | G6PD/6PGD ratio method | 1.00-2.30 |

G6PD, glucose-6-phosphate dehydrogenase.
